# The Risk of Pulmonary Embolism in Patients With Pemphigus: A Population-Based Large-Scale Longitudinal Study

**DOI:** 10.3389/fimmu.2019.01559

**Published:** 2019-07-24

**Authors:** Khalaf Kridin, Mouhammad Kridin, Kyle T. Amber, Guy Shalom, Doron Comaneshter, Erez Batat, Arnon D. Cohen

**Affiliations:** ^1^Department of Dermatology, Rambam Health Care Campus, Haifa, Israel; ^2^Sackler School of Medicine, Tel Aviv University, Tel Aviv, Israel; ^3^Department of Dermatology, University of Illinois at Chicago, Chicago, IL, United States; ^4^Siaal Research Center for Family Medicine and Primary Care, Faculty of Health Sciences, Ben-Gurion University of the Negev, Be'er Sheva, Israel; ^5^Department of Quality Measurements and Research, Chief Physician's Office, Clalit Health Services, Tel Aviv, Israel

**Keywords:** pemphigus, pulmonary embolism, risk, epidemiology, thromboprophilaxis

## Abstract

Growing evidence suggests that inflammation may pose an atypical risk factor for pulmonary embolism (PE), as it drives venous thrombosis via several pathways. The increased risk of PE in several autoimmune diseases has lent weight to this concept. However, the relative risk of PE among patients with pemphigus has not yet been established. We aimed to examine the risk of PE in patients with pemphigus. A large-scale population-based longitudinal cohort study was conducted to evaluate the relative risk (RR) of PE among 1,985 patients with pemphigus relative to 9,874 age-, sex-, and ethnicity-matched control subjects. A multivariate Cox regression model was utilized. The incidence of PE was 3.0 (95% CI, 2.2–4.0) and 1.2 (95% CI, 1.0–1.5) per 1,000 person-years among patients with pemphigus and controls, respectively. The period prevalence of PE corresponding to the study period was 2.2% (95% CI, 1.6–2.9%) among cases and 0.9% (95% CI, 0.7–1.1%) among controls. Patients with pemphigus were twice as likely to develop PE as compared to control subjects (adjusted RR, 1.98; 95% confidence interval [CI], 1.29–3.04). The highest PE risk was observed during the 1st year following the diagnosis of pemphigus (adjusted RR, 3.55; 95% CI, 1.78–7.09) and decreased over time. The increased risk was robust to a sensitivity analysis that included only cases managed by pemphigus-related systemic medications (adjusted RR, 1.82; 95% CI, 1.11–2.98). In conclusion, pemphigus is associated with an increased risk of PE, particularly during the 1st year of the disease. An awareness of this risk should be increased, additional precipitating factors for PE should be avoided, and thromboprophylaxis may be evaluated in high-risk patients. Further research is required to establish this risk.

## Introduction

Pulmonary embolism (PE) is a life-threatening cardiovascular and cardiopulmonary condition associated with substantial burden (1). The estimated annual incidence rate of PE ranges from 0.15 to 1.0 cases per 1,000 populations (1). It is characterized by high mortality rates that may exceed 15% within the first 3 months following diagnosis (2), accounts for 5–10% of deaths among hospitalized patients, and is the most common preventable cause of inpatient death (3).

Growing evidence suggests that inflammation may pose an atypical risk factor for PE, as it drives venous thrombosis via several pathways (4). The increased risk of PE in several autoimmune diseases has lent weight to this concept (5). However, the relative risk of PE among patients with pemphigus has not yet been established. Additional prominent risk factors are institutionalization, malignancies, trauma, congestive heart failure, central venous catheter, or pacemaker placement (6).

The aim of the current study was to investigate the risk of development of PE using a large-scale population-based longitudinal cohort study.

## Methods

### Study Design and Data Source

We conducted a longitudinal cohort study to examine whether patients with pemphigus are at increased risk to develop PE. The current study utilized data from the Clalit Healthcare Services (CHS) computerized database. Patient data was retrieved using data mining techniques.

The CHS database is the largest managed care organization in Israel, insuring approximately 4,400,000 enrollees in 2016. It is an inclusive computerized database with continuous real-time input from medical, pharmaceutical, and administrative operating systems. The validity of diagnoses in this database, which are grounded on hospital and primary care physicians and specialists reports, was previously identified to be of high validity (7).

### Study Population

We identified all individuals with an incident diagnosis of pemphigus between 2004 and 2014 using the CHS database. Patients were defined as having pemphigus when there was a diagnosis of pemphigus documented at least twice in the medical records as registered by a physician in the community, or when pemphigus was listed in the diagnoses of discharge letters from a hospital. A control group of up to five control subjects per case was selected and matched randomly by age, sex, and ethnicity. Age matching was based on the exact year of birth (1-year strata). Controls were verified to be alive and contributing data to CHS on the date of the diagnosis of the matched case. The date of enrollment of the control participant was identical to the date of diagnosis of the matched case. The study population was followed until December 31, 2016 or until emigration, death, or occurrence of the primary endpoint, whichever occurred first.

### Statistical Analysis

Incidence rates of PE were calculated for both pemphigus patients and controls and expressed as the number of events per 1,000 person-years.

Analysis of PE risk was performed in a multivariate Cox regression model using calendar time as the time scale and adjusting for possible confounders, including age, sex, malignancy, smoking, peripheral vascular disease, congestive heart failure, ischemic heart disease, history of cerebrovascular accident, hyperlipidemia, diabetes mellitus, hypertension, obesity, chronic obstructive pulmonary disease, and exposure to systemic corticosteroids. All of these variables were handled as binary variables in the analysis, with age being classified as either > or ≤ the median age of patients (72 years). The Cox regression model was used to compute hazard ratios of PE as a measure of relative risk (RR).

A 2-sided *P*-value < 0.05 was considered statistically significant. Data management and statistical analyses were performed with the use of SAS software version 9.4 (SAS Institute, Cary, NC, USA).

## Results

The total sample included 11,859 eligible patients, 1,985 of whom had pemphigus, and 9,874 were age-, sex-, and ethnicity-matched control subjects. Descriptive characteristics of the study participants are shown in Table 1. The mean (±SD) age at presentation of cases and enrollment of controls was 72.1 ± 18.5, and 59.8% of participants were female. The ethnic and the socioeconomic structure of the two groups was comparable. Comorbidity rates, as measured by the Charlson index, were higher in cases, with 1,059 (53.4%) patients having severe comorbidity compared to 4,055 (41.1%) in controls (*P* < 0.001; Table 1).

**Table 1:** Descriptive characteristics of the study population.

**Characteristic**	**Patients with pemphigus (*N* = 1,985)**	**Controls (*N* = 9,874)**	***P*-value**
**Age, years**			
Mean ± SD	72.1 ± 18.5	72.1 ± 18.5	1.000
Median (range)	77.4 (0–103.0)	77.4 (0–103.1)	
Male sex, *N* (%)	797 (40.2%)	3,962 (40.1%)	0.934
**Ethnicity**, ***N*** **(%)**			
Jews	1,805 (90.9%)	8,866 (89.8%)	0.136
Arabs	180 (9.1%)	1,008 (10.2%)	
BMI, kg/m^2^ (Mean ± SD)	27.7 ± 6.6	27.9 ± 6.6	0.355
Smoking, *N* (%)	510 (25.7%)	2,758 (27.9%)	0.045
**SES**, ***N*** **(%)**			
Low	634 (31.9%)	3,249 (32.9%)	0.386
Intermediate	830 (41.8%)	4,263 (43.2%)	0.250
High	423 (21.3%)	2,217 (22.5%)	0.241
**Charlson comorbidity score**, ***n*** **(%)**			
None (0)	344 (17.3%)	2,636 (26.7%)	<0.001
Moderate (1–2)	582 (29.3%)	3,183 (32.2%)	0.011
Severe (≥3)	1,059 (53.4%)	4,055 (41.1%)	<0.001

*N, Number; SD, standard deviation; BMI, body mass index; SES, socioeconomic status*.

The median follow-up time was 6.8 years (range, 0–13.0 years) for patients with pemphigus and 6.9 years (range, 0–13.0 years) for control subjects. The total follow-up time was 13,936.3 person-years for patients with pemphigus and 70,550.6 person-years for controls. The incidence of PE was 3.0 (95% CI, 2.2–4.0) and 1.2 (95% CI, 1.0–1.5) per 1,000 person-years among patients with pemphigus and controls, respectively. The period prevalence of PE corresponding to the study period was 2.2% (95% CI, 1.6–2.9%) among cases and 0.9% (95% CI, 0.7–1.1%) among controls.

### The Risk of PE in Patients With Pemphigus

The crude risk of PE was over 2-fold higher in patients with pemphigus than in the matched control group (RR, 2.47; 95% CI, 1.71–3.57). The risk of PE was elevated in both male (RR, 2.81; 95% CI, 1.46–5.41) and female (RR, 2.33; 95% CI, 1.49–3.65) patients.

We then conducted multivariate analysis to identify the predicting factors for PE. After adjusting for several confounding factors, pemphigus emerged as an independent significant risk factor for incident PE (adjusted RR, 1.98; 95% CI, 1.29–3.04; Table 2). Peripheral vascular disease (PVD; adjusted RR, 1.78; 95% CI, 1.08–2.92), smoking (adjusted RR, 1.52; 95% CI, 1.01–2.29), and the administration of systemic corticosteroids (adjusted RR, 1.59; 95% CI, 1.07–2.36) were also significantly associated with the development of PE (data not shown).

**Table 2:** The risk of pulmonary embolism in patients with pemphigus stratified by follow-up time.

**Duration of follow-up**	**Crude RR**	**95% CI**	***P*-value**	Adjusted RR^*^	**95% CI**	***P*-value**
Any duration	2.47	1.71–3.57	< 0.001	1.98	1.29–3.04	0.002
<1 year	5.04	2.79–9.12	<0.001	3.55	1.78–7.09	<0.001
2–5 years	1.58	0.87–2.87	0.137	1.25	0.63–2.47	0.518
6–10 years	1.79	0.71–4.53	0.222	1.89	0.64–5.58	0.247

*RR, risk ratio; CI, confidence interval*.

* *Adjusting for age, sex, malignancy, smoking, peripheral vascular disease, congestive heart failure, ischemic heart disease, history of cerebrovascular accident, hyperlipidemia, diabetes mellitus, hypertension, obesity, chronic obstructive pulmonary disease, and exposure to systemic corticosteroids*.

Disaggregation by follow-up time revealed that the highest risk of PE was within the 1st year following the diagnosis of pemphigus (adjusted RR, 3.55; 95% CI, 1.78–7.09). The adjusted RR decreased over time to 1.25 (95% CI, 0.63–2.47) at 2–5 years, and to 1.89 (95% CI, 0.64–5.58) at 6–10 years after diagnosis (Table 2).

### Sensitivity Analysis

We then performed a sensitivity analysis including only patients with pemphigus who were prescribed one of the following pemphigus-related treatments: systemic corticosteroids; adjuvant immunosuppressants; azathioprine, mycophenolate mofetil, cyclophosphamide; or rituximab. The increased risk for PE retained its statistical significance both in univariate (RR, 2.60; 95% CI, 1.76–3.86) and multivariate analyses (adjusted RR, 1.82; 95% CI, 1.11–2.98).

## Discussion

This large-scale population-based study provides evidence that patients with pemphigus are twice as likely to develop PE than matched control subjects. This increased risk was most pronounced within the 1st year following the diagnosis of pemphigus and thereafter declined over time to lose its statistical significance.

### Previous Literature

A growing body of evidence has suggested that several autoimmune disorders are significantly associated with an increased risk of PE, including rheumatoid arthritis (5, 8), systemic lupus erythematosus (5, 9), dermatomyositis/polymyositis (5), type 1 diabetes mellitus (10), inflammatory bowel disease (5, 11), Behçet‘s disease (5, 12), celiac disease (5, 13), Sjögren's syndrome (5), systemic sclerosis (5), granulomatosis with polyangitis (5, 14), and polyarteritis nodosa (5).

Data regarding the association of pemphigus with PE is extremely limited. In their hospital-based uncontrolled cohort study of 172 patients with pemphigus, Leshem et al (15) found that 5 (2.9%) patients developed PE within a median duration of 4 months following the diagnosis of pemphigus. The external validity of this study was hindered by the lack of a matched control group; this interfered with the evaluation of the relative risk of PE in patients with pemphigus relative to the reference population (15). In a British population-based study examining the risk of venous thromboembolism (VTE) among patients with various autoimmune diseases, an elevated VTE risk was observed in a cohort of inpatients individuals with pemphigus and pemphigoid grouped together (16). Neither the PE-specific nor the pemphigus-specific outcome measures had been depicted in this study (16). Taken together, the actual burden of PE in pemphigus is yet to be established.

### The Interpretations of the Findings

The mechanism underlying the increased PE risk in pemphigus had not been clearly defined. However, there is evidence that systemic inflammation, which exists in pemphigus as well as in other autoimmune diseases, potentiates venous thromboembolism by up-regulating procoagulants, down-regulating anticoagulants, and suppressing fibrinolysis (4). The cross-talk between inflammation and coagulation amplifies and maintains the activation of both systems (17). Nonetheless, a study of 23 patients with pemphigus vulgaris and 10 controls found that the levels of D-dimer, plasma prothrombin fragments F1+2, and serum tissue factor were within the normal limits, both in active and in remittent disease (18). The use of oral corticosteroids both in low and high dosages carries an elevated risk of PE, as they promote hemostasis by increasing the levels of fibrinogen and clotting factors (19). Given that corticosteroids remain the mainstay of treatment in pemphigus and are administered in the vast majority of patients, they may embody a main risk factor of PE in pemphigus. Our multivariate analysis found that administration of corticosteroids was associated with a 1.6-fold increased PE risk, and thus substantiates this hypothesis.

Hospitalization was identified as a notable precipitating factor for the development of subsequent PE (20), and it is very frequent among patients with pemphigus in several healthcare systems (21, 22). Leshem et al (15) suggested that pemphigus patients may experience immobility stemming from corticosteroid-induced myopathy and fractures due to corticosteroid-induced osteoporosis and are, therefore, more vulnerable to develop PE (16). The high prevalence of infections among patients with pemphigus may be another putative explanation for the observation (23), as infections significantly predispose to PE (24).

### The Implication of the Findings

Of great interest, the highest risk of PE occurred within the 1st year following the diagnosis of pemphigus and declined thereafter. This finding is in accordance with the observation of Leshem et al (15), who reported that four of the five PE events occurred during the 1st year of the disease. Correspondingly, a nationwide Danish large-scale study demonstrated that the risk of PE was prominently increased in the 1st year after the initial diagnosis of 33 autoimmune diseases, and decreased subsequently as the duration of follow-up became longer (5). The interpretation of this phenomenon may lie in the fact that the inflammatory load of pemphigus as well as the exposure to treatment and its complications is maximal during the 1st year of diagnosis and decreases over time (5, 15). Since inflammation promotes several procoagulant pathways, effective treatment leading to the suppression of the inflammatory state could, therefore, decrease the risk of PE over time (5). Based on our findings, we recommend close monitoring of these patients mainly during the 1st year following diagnosis.

The elevated PE hazard in pemphigus begs the question of whether or not thromboprophylaxis is favorable. The benefit of this intervention should be weighed against the risk of bleeding, particularly in patients frequently managed by high-dose corticosteroids for extended periods of time. The current guidelines recommend thromboprophylaxis solely for hospitalized patients with a high underlying risk of thrombosis (25). Our multivariate analysis, however, showed that pemphigus by itself is an independent risk factor of PE, regardless of the existence of other precipitating variables.

### Strengths and Limitations

Our study is a population-based controlled study aiming to estimate the risk of PE in pemphigus. The study provides a standardized and large cohort size, which gives sufficient power to exclude chance as the basis for the findings and to minimize the probability of selection bias. Our study, however, has some limitations that should be addressed. Since the CHS registry was originally designed for clinical purposes and not for fulfilling formal disease criteria, validation of the diagnoses of pemphigus and PE is lacking. Nevertheless, specialists are required to affirm the diagnoses, which are then added by primary care physicians into the database. Previous studies based on the CHS database have shown high reliability of the data (26). In addition, data concerning the clinical characteristics and the severity of the two conditions could not be obtained for the current study. The existence of surveillance bias stemming from the increased medical scrutiny for patients with pemphigus could not be definitely eliminated; however, it is less probable in a devastating outcome like PE. Although we have adjusted for various potential confounding conditions and performed a sensitivity analysis, a potential risk for residual confounding cannot be thoroughly excluded. Misclassification of the outcome, if existed, is most likely non-differential, thus leading to underestimation of effect sizes. Additionally, we could not rule out the probability that genetic coagulopathies are more prevalent among patients with pemphigus based on our dataset. However, this hypothesis was refuted in an Italian study that observed neither elevated peripheral levels of D-dimer and tissue factor (TF) nor increased TF immunoreactivity in skin specimen of 23 patients with pemphigus vulgaris (18).

In conclusion, our study demonstrates that patients with pemphigus have a 2-fold increase in risk to develop PE, particularly during the 1st years following the diagnosis. Further observational studies are necessary to establish this association in other populations. The present findings may increase the awareness of PE in patients with pemphigus, and encourage both clinicians and patients to avoid additional risk factors of PE. Thromboprophylaxis may be considered in patients for whom the underlying risk of PE is especially high, particularly in the 1st year after diagnosis.

## Data Availability

All datasets generated for this study are included in the manuscript and/or the supplementary files.

## Author Contributions

All authors listed have made a substantial, direct and intellectual contribution to the work, and approved it for publication.

### Conflict of Interest Statement

The authors declare that the research was conducted in the absence of any commercial or financial relationships that could be construed as a potential conflict of interest.
